# Modern-type depression and web-based psychopathology in a cohort of Italian university students

**DOI:** 10.1192/j.eurpsy.2022.627

**Published:** 2022-09-01

**Authors:** L. Orsolini, S. Bellagamba, G. Longo, S. Tempia Valenta, V. Salvi, U. Volpe

**Affiliations:** 1 Unit of Clinical Psychiatric, Polytechnic University of Marche, Ancona, Italy, Department Of Neurosciences/dimsc, Ancona, Italy; 2 Unit of Clinical Psychiatry, Department Of Neurosciences/dimsc, Polytechnic University Of Marche, Ancona, Italy; 3 Polytechnic University of Marche, Department Of Clinical Neurosciences/dimsc, School Of Medicine, Unit Of Psychiatry, Ancona, Italy; 4 Unit of Clinical Psychiatry, Polytechnic University of Marche, Ancona, Italy, Department Of Neurosciences/dimsc, Ancona, Italy

**Keywords:** digital addiction, Modern Type Depression

## Abstract

**Introduction:**

Hikikomori represents the severe social withdrawal condition of the so-called ‘modern type-depression” (MTD). Digital addictions, including Internet addiction (IA), Internet gaming disorder (IGD) and smartphone addiction, have been associated with MTD and Hikikomori.

**Objectives:**

This is a post-hoc study aimed at assessing digital addictions in a cohort of university students with a positive screening for MTD and Hikikomori.

**Methods:**

A cross-sectional web-based survey was conducted by administering the Hikikomori Questionnaire (HQ-11), Internet Addiction Test (IAT), Internet Gaming Disorder Scale-Short-Form (IGDS9-SF) and the Smartphone addiction scale-Short Version (SAS-SV).

**Results:**

Among 1,148 respondents, a significant association was found between the HQ-11 scale and the DASS-21 total score (r=0.434). The HQ-11 positively correlated with IAT, IGDS9-SF and SAS-SV (r=0.329; r=0.292 and r=0.205 respectively).

**Conclusions:**

Digital addictions appear to be widely diffuse among university students positive to the Hikikomori and MTD screening. Further longitudinal studies are needed to weight and balance the potential consequences of digital tools in Hikikomori subjects.

**Disclosure:**

No significant relationships.

